# Does it fit? – Trainability of affordance judgments in young and older adults

**DOI:** 10.1371/journal.pone.0212709

**Published:** 2019-02-28

**Authors:** Lisa Finkel, Simone Engler, Jennifer Randerath

**Affiliations:** 1 Department of Psychology, University of Konstanz, Konstanz, Germany; 2 Lurija Institute for Rehabilitation Science and Health Research, Allensbach, Germany; Tokyo Daigaku, JAPAN

## Abstract

Will I fit into the overcrowded subway? Advanced aging can change our abilities associated with accurately judging the fit between perceived environmental properties and our own actual physical capabilities (affordance judgments). Two experimental studies examined the effects of aging and trainability in affordance judgments. Participants were asked to decide whether their hand fits into a given opening (Aperture Task). We used a detection theory approach to evaluate different judgment characteristics. Study 1 demonstrated that older (*N* = 39) compared to younger adults (*N* = 39) produced rather conservative judgments, but did not differ in perceptual sensitivity. Distributions of Hit and False-Alarm rates, as well as risk-perception statements (DOSPERT questionnaire), indicated a heightened concern about potential consequences of misjudgments in older adults. In Study 2, 20 younger and 22 older adults were trained by actually trying to fit their hand into each presented opening. Training included acoustic, haptic and visual feedback. Compared to pre-training, both groups demonstrated significant increases in accuracy when assessed post-training and after a one-week follow-up. While younger adults improved in perceptual sensitivity in post-training as well as in follow-up, the older group adjusted their tendency towards less conservative judgments in both following sessions. Our results are consistent with affordance models that propose a complex and dynamic interplay of different neural processes involved in this skill. Future studies are needed to further elucidate that interplay and the trainability of affordance judgments.

## Introduction

When navigating through our environment, we recurrently make judgments upon whether a certain action is possible or not. The ecological *theory of affordances* by Gibson states that an important aspect of this ability is that “the information to specify the utilities of the environment is accompanied by information to specify the observer himself, his body, legs, hand, and mouth*”* [1]. The central idea is that propertied substances and surfaces afford actions, meaning that they offer the potential for certain actions or constraints. At the same time, affordances need to be considered relative to the individual’s action capabilities and therefore are unique to the individual. Thus, affordance judgments are based on the fit between perceived environmental properties and one’s own physical capabilities

Many studies on affordance perception focused on object and tool manipulation such as functional tool use or grasping (for a discussion on “object-related” affordances see references: [2–4]). The current study on affordance perception instead focuses on judging “actor-related” affordances, i.e. judgments upon affordances that emerge from the fit of the actor’s capabilities and environmental properties. (The terms “object-related” and “actor-related” have been introduced in a recent review on actor-related affordance judgments [5]). Research has shown that healthy young adults are quite able to perform appropriate affordance judgments, such as when reaching for objects [6–8], passing the body through apertures [9, 10], stepping across obstacles [11], standing upright on inclined surfaces [12, 13], and fitting a hand into an aperture [14, 15].

In the past 10 years, research has moved into examining training interventions with the goal of improving performance in affordance judgments. For example, it has been shown that individuals can improve their affordance judgments subsequent to action practice [16–19]. Moreover, the combination of actually executing the particular task and additionally receiving feedback on the accuracy of preceding judgments seems to be particularly effective. For example, training interventions have been effective, when fitting the hand through apertures [15], when vertically reaching for objects [15], or when reaching overhead for objects [20]. Further research demonstrated, that experiencing both successful and unsuccessful trials in training intervention (e.g. when walking or squeezing through some apertures that are smaller and some that are wider as minimum passable width) was necessary for improving affordance judgments [21, 22].

There is little experimental research on potential age-related changes in such actor-related affordance judgments. That is surprising considering that the body and, associated therewith, actor-related action capabilities change throughout the lifespan [23, 24]. For instance, when judging actor-related affordance judgments, age-related bodily alterations have to be considered. Further, capabilities like flexible information processing [25, 26], motor reaction capacity and speed [27, 28] and visuospatial abilities [29] also decline with advancing age in healthy individuals; those traits might also effect appropriate affordance perception. The existing research on age-related effects within the scope of affordance judgments demonstrates ambiguous results in that older adults either showed similar [30–32] or worse judgment performance compared to younger adults [33, 34].

Gibson’s ecological theory of affordances [35] claimed that affordance perception is a process of perceiving an object that has ecological value. Because affordances are properties referring to the observer, Gibson stated that “any substance, any surface, any layout has some affordance for benefit or injury to someone” ([35], p. 140). He further stated, that affordances themselves are no properties of the observer’s experience ([35], p. 137), because their sole existence is independent from the observer. Other authors pointed out that the ability to make judgments based on affordance perception, however, may be dependent or influenced significantly by experience and learning ([36], p. 219). Dependent on the observed situation and prior experience the actor may apply a rather conservative or riskier judgment tendency for a planned behavior. Thus, affordance judgments may involve the processing of both, on-line perceived environmental properties as well as an experience-based judgment criterion. As part of the developments of a broadening affordance concept that additionally takes neural processes into account, newer approaches [37–39] integrated these variables into a dynamic motor-cognitive model for action selection. For example, in Cisek’s ‘affordance competition’ model judgments upon possible actions are based on a competition between affordances that for example is influenced by attentional processes, anticipated consequences, and biases that may come from many sources in a complex bilateral brain network. Considering different sources of information into account may support adaptive behavior. In line with such an ecological perspective, affordance judgments appear to be task and setting specific. When examining older adults’ ability to accurately judge their affordances, studies report ambiguous results. Some reflect realistic judgments and others demonstrated tendencies to either over- or underestimate one’s own capabilities. For example, in some incidences, older adult perceptual judgments matched their actual action capabilities when deciding upon their largest possible riser height for stair climbing [30, 31], or when judging maximal reach while standing and leaning forward [40]. In contrast, studies evaluating judgments on street crossing capabilities reported overestimations in older adults, who selected insufficiently large gaps in oncoming traffic [33] and misjudged the time that was needed to safely cross a street [34]. Other studies stressed tendencies towards underestimations, for example, when walking through doorways [41].

With the goal to further elucidate aspects of affordance judgments and particularly trainability in healthy young and older adults, we implemented two studies that evaluated judgment performance and tendencies associated with hand-fitting aperture tasks. In the first study, we examined older versus young healthy adults’ ability to accurately judge their actor-related affordances while judging whether their hand fits into an opening. We used a standardized Aperture Task that had been proven to be applicable to different populations [15, 42]. To determine the relationship between affordance judgments and the perception of relevant body references, we added a hand-size estimation-task.

In the second study, we assessed older versus young healthy adults’ trainability while experiencing and receiving feedback in the Aperture Task. We examined whether training improves affordance judgments after one week.

In order to have a more elaborate assessment, we applied the detection theory approach for statistical analysis and thereby considered the participant’s perceptual sensitivity (discriminability index d’, i.e. ability to discriminate between a fit and a non-fit) as well as their response tendencies (criterion c: rather conservative versus liberal) as main variables [43–45]. Data from previous studies that assessed the use of the paradigm in young adults [15] and the effect of stroke on affordance perception respectively [42], seems to indicate that younger and older adults perform on an equal perceptual sensitivity level in the Aperture Task, however, older adults tend to judge more conservatively. In line with the assumption that affordance perception engages a complex motor-cognitive network comprising different factors, our earlier work with healthy young adults demonstrated that affordance judgment performance is correlated with the ability to accurately perceive size [15].

Based on indications from previous work, for Study 1, we predicted that older adults would perform on a similar level in perceptual sensitivity compared to younger adults, but older adults would apply a more conservative judgment tendency. Furthermore, size perception would correlate with performance in the Aperture Task.

In Study 2, we focused on trainability. Since there are a few studies demonstrating the positive effects of training, we expected judgment performance, measured by judgment accuracy and perceptual sensitivity, to increase after one single training intervention in both groups. We also assumed that training effects would endure over the one-week follow-up. Since our previous study results revealed that participants seem to choose a rather conservative judgment tendency in the Aperture Task [15, 42], we further hypothesized a training-induced adjustment of the tendency towards a less conservative judgment tendency particularly in older adults.

## General materials and methods

This project was approved by the ethical committee of the University of Konstanz and conducted in accordance with the Declaration of Helsinki. All participants provided informed written consent and received financial or study credit compensation. One participant was tested shortly before turning 18 years old (legal adult age according to German law), for this person written consent was additionally obtained from the parents.

The present investigation included two studies conducted within 9 days: an initial diagnostic study and a training study. For Study 1, performance in the Aperture Task was measured in one session (day 1); 78 individuals took part. Approximately half of the total sample (*n* = 42) subsequently participated in Study 2 which involved two more sessions: one session included a repeated assessment and the training intervention (day 2). A follow-up session was administered after one week to test possible lasting effects of training (day 7–9; depending on the temporal availability of participants).

First, we will describe the general methods including materials and measurement procedures which were adapted from previous work [15]. Subsequently, we will present specific information on sample characteristics and data analyses as well as the results per study.

### Participants

Participants were recruited between May 2017 and May 2018 by announcement and postings at a university setting and in local municipal facilities and buildings. Older adult participants were additionally recruited at retirement housings and via activity programs offered for older adults by the local community German Red Cross. All participants were right-handed (diagnosed with lateralization quotient ≥ 60; [46]), had a normal or corrected-to-normal vision and were naïve to the specific goals of the study. The present sample consisted of participants with low to medium (school education, vocational training) and high (university and post-graduate education) levels of education. None of the participants reported a history of psychiatric or neurologic disorders. We further implemented a cognitive screening test to preclude the selection of any condition of dementia or mild cognitive impairment (DemTect; [47–49]). The test included a word list, a number transcoding task, a word fluency task, digit span reverse, and delayed recall of the word list. According to the test guidelines and the respective age group, participants’ raw scores were transformed into test scores. For participants that scored below the cut-off test score (13 points), mild cognitive impairment (9–12 points) or dementia (0–8 points) can be suspected. Participants had to achieve the minimum 13 points to be included in the present studies.

In total, 78 individuals fulfilled inclusion criteria and took part in Study 1. Of that total, 42 participated in Study 2 which involved a training as well as a follow-up session. The samples for Study 1 and Study 2 will be further specified in the respective study’s methods section.

### Material

**Fig 1:**
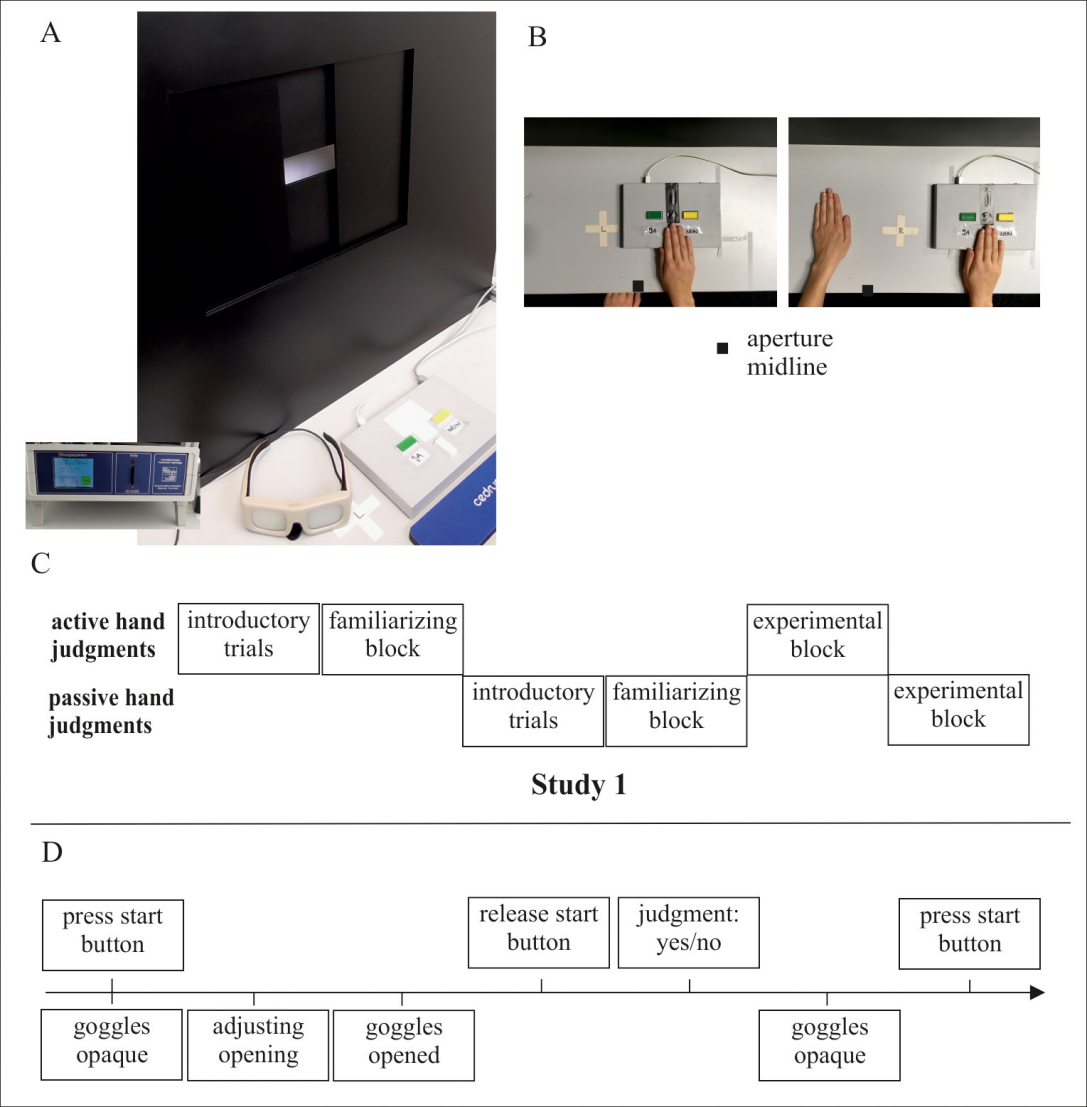
Experimental setting and procedure. (A) Experimental setting including Aperture apparatus, goggles (here: opaque), button-pad and the control device coding trial sequence and controlling the motor. The example setting is prepared for a person using the right hand for button pressing (B). The first image shows the button-pad being positioned on top of the stimulus mark for the right hand (hidden cross with “R”; referring to right hand as stimulus). The participant judges whether the right hand fits into the opening. The second image shows the button-pad set aside. The participant positions the left hand on top of its stimulus-mark (hidden cross with “L”; referring to left hand as stimulus) and judges whether the left hand may fit into the aperture 2. Thus, the stimulus-mark indicated where the participant has to position the hand to be judged. The response is indicated by pressing the yes or no button with the right hand. Vice versa, for active left-hand judgments and passive right-hand judgments, positioning of the button-pad was mirrored along the aperture midline. Chart (C) delivers an overview of the procedure in Study 1. Chart (D) an outlined sequence of one judgment trial.

The hand aperture apparatus was custom built with a rectangular opening centrally placed so height and width could be manipulated to adjust for individual body size. Experimental data were coded with SuperLab 5 Software (provided by Cedrus). Trial protocol related adjustments for the rectangle’s width were programmed and regulated by a computer-controlled motor. Two hand tasks were performed: the affordance judgment task (Aperture Task) and the size perception task. Throughout the experiment, participants wore Plato-goggles (Translucent Technologies Inc.) that could be switched between transparent and opaque and thus allowed to control vision and prevent visual feedback when necessary. Fig 1A illustrates the experimental setting.

### Tasks and procedures

#### Measurements

Each session started out with measuring the maximum width and the height of the participant’s hands (typically at the transition of the proximal phalanges and metacarpal bones). Hands were held flat in the aperture with fingers closely spaced, and hand size measurements were taken by closing the opening tightly around the hand’s widest part. The height or vertical opening size was set to the thickness of the participant’s individual hand (i.e. height measured from the palm to the dorsum of the hand). For the entire measuring procedure, participants received haptic feedback while visual feedback was avoided by shutting the Plato-goggles.

#### Aperture task

In the affordance perception task, participants judged whether they could fit the widest part of their hand through a given horizontal aperture. Participants were asked to respond as accurately as possible. Presented aperture sizes were based on the participant’s actual hand width and the addition or subtraction of a fixed set of increments (hand width +/- 0, 2, 4, 8, or 16 mm) which were presented in a fixed randomized order. The 0-trial reflected the participant’s measured hand width (i.e. minimum aperture size the hand fitted through). One filler trial (smaller opening: hand width either minus 20, 30 or 40mm) per set of increments was added to achieve a balance between yes- and no-trials (i.e. equal number of correct “yes” responses for the openings 0, 2, 4, 8, 16 mm and correct “no” responses for the openings -2, -4, -8, -16mm and the filler trial). Participants started with introductory trials with extremely small or large openings including 6 trials in order to accustom participants to task procedures. Furthermore, data from a previous study [15] indicated that during the first few judgment trials, formation of a stable judgment tendency might occur. Thus, participants performed a familiarizing block of 20 trials before the experimental blocks. The experimental diagnostics block consisted of 3x9 openings, plus 3 filler trials, plus 2 extreme openings. Trials were blocked per hand to be judged, and therefore either involved judgments for the right or the left hand. An illustration of the procedure is depicted in Fig 1C. In each block, the analyzed increments (hand width +/- 0, 2, 4, 8, or 16 mm) were presented with equal frequency. Participants indicated their judgments by pressing a specified “yes” or “no” button on a button-pad (Cedrus, RB540). Before each block, the participant was reminded of what hand to use. The hand was positioned visible on a stimulus-mark. The stimulus-mark was shifted from the aperture’s midline (8 cm) to avoid direct alignment strategies.

Although this is not specifically relevant for the current study sample with healthy young and older adults, the entire experimental design has been designed to be applied across various samples including those who may have unilateral motor deficits or visuo-spatial deficits (e.g. stroke patients). Furthermore, stroke patients (or any other participants with motor disabilities due to accidents, arthritis, multiple sclerosis, etc.) may be restricted to use the least affected hand for indicating their judgments with a button press response. Also they may not be able to change the hand’s position frequently. Thus, judgment trials for the left and the right hand were presented in a blocked manner and participants only used one assigned hand for button presses. The hand that pressed the button was named the “active hand”. If participants made judgments for their button-pressing active hand, the button-pad was set on top of the stimulus-mark and the passive hand remained in the participant’s lap (Fig 1B left). If the passive hand was judged, the button-pad with the active hand was moved 20 cm toward the outer edge of the apparatus to allow for the passive hand to be positioned on the stimulus-mark (Fig 1B right). Thus, the hand to be judged was positioned visible in front of the participant. Approximately half of the group used their left hand for indicating responses (left hand = button-pressing active hand), the other half used their right hand (right hand = button-pressing active hand).

Further, to consider a potential visuo-spatial hemi-neglect when studying affordance judgments in stroke patients, the shift was yoked with the group either toward left hemispace (in case of a left button-pressing hand) or right hemispace (in case of a right button-pressing hand).

Participants always started to make their affordance judgments for the assigned active, button-pressing hand. The sequence of one judgment trial is exemplified in Fig 1D. In Fig 1A and 1B it can be seen that the stimulus-mark (taped white cross on the table) was placed slightly sideward towards the active hand in order to avoid direct alignment strategies relative to the opening.

#### Size-estimation task

In order to evaluate the impact of the participants’ ability to estimate the hand’s horizontal size, a control task was conducted following each affordance-judgment session. While the vertical height remained set to the height of the individual’s hand, the horizontal width was either gradually increased or decreased. For half of the trials, horizontal width was increased from 0mm, while the other half openings were gradually decreased starting at a 200mm opening. Participants were instructed to say “stop” when the gradually adjusted opening width had the same size as the widest part of their hand. They were allowed to correct their initial estimation by saying “smaller” or “wider” until being satisfied with the final position. Participants made the same number of judgments for each hand.

#### Domain-specific risk-taking scale

In order to assess a potential link of an applied judgment tendency in the Aperture Task and participant’s risk-behavior, we implemented the German version of the Domain-Specific Risk-Taking scale (DOSPERT; [50, 51]). The DOSPERT scale includes 30 items grouped in five content domains. In a first part, participants rated the likelihood that they would engage in domain-specific risky activities on a 7-point Likert Scale (highly unlikely to highly likely), afterward participants rated the magnitude of these risks (absolutely no risk to very high risk). In the analyses, we focused on those items that belong to the “health and safety” subdomain (e.g. “Riding a motorcycle without a helmet” or “Sunbathing without sunscreen”) since the subdomain corresponds best to actor-related judgments. For 14 young and one older adult, no DOSPERT data was available.

### Data analysis

Behavioral data were analyzed with SPSS 25 (IBM). The average number of missing trials, e.g., due to no recordable response by the participants was very low (< 1%). Exact *p* values were reported 2-tailed (*p* ≤ .05). Normality was assessed by screening normal probability plots and with Shapiro-Wilk Test. Information on general judgment accuracy (percent of correct judgments) was analyzed.

In addition, detection theory variables were extracted [15, 42]. With the aim to facilitate interpretation and prediction of potential consequences of misjudgments, information about two independent measures is provided: judgment tendency and perceptual sensitivity. Calculations of perceptual sensitivity (discriminability index d-prime) and judgment tendency (criterion c) are based on Hit and False-Alarm rates [43–45]. This approach has considerable advantages since aside from describing the accuracy of judgments, it additionally provides important information on the type of conducted errors (miss: indicating “no” even when the hand would fit through the aperture; false-alarm: saying “yes” even when the hand does not fit through the aperture).

Whereas the False-Alarm rate is calculated by the ratio of the number of negative events wrongly categorized as positive (i.e. indicating “yes” in trials, the hand actually does not fit through the given opening) and total number of actual negative events, the Hit rate depicts the ratio of the number of positive events successfully categorized as positive (i.e. indicating “yes” in trials). That is, the hand actually fits through the given opening. Please note that discriminability index (d-prime) and criterion (c) are independent measurements [45, 52]. The discriminability index (d-prime) measures the participant’s ability to discriminate a fit from a non-fit. The more sensitive the participant is at discriminating, the larger the d-prime value will be. The perceptual sensitivity was calculated using the following formula: *d'* = Z(Hit rate)—Z(False-Alarm rate). The participant’s judgment tendency is indicated by the criterion (c): a positive c value represents a rather conservative judgment tendency (i.e. respond “no” more often than the ideal observer), while a negative c value is associated with increasingly liberal judgments. The judgment tendency was calculated using the following formula: *c* = -.5*[Z (Hit rate)+Z(False-Alarm rate)].

Even though the two main measures d-prime and criterion c are calculated on the basis of Hit and False-Alarm rates, additional information on Hit and False-Alarm rates and their distribution was provided. The aim was to facilitate comprehension of d-prime and criterion measures and thereby the interpretability of the behavioral results. Further, it would be interesting to examine whether both rates change as a result of training. For example, would a potential increase of yes-responses affect both, Hit and False-Alarm rates, or only one of these two variables? Either option would lead to a less conservative response tendency.

## Study 1

In Study 1, we aimed at examining potential advanced aging effects in performing affordance judgments. This portion of the investigation comprised one session lasting approximately one and a half hours.

### Study 1 methods

#### Sample

Study 1 included 39 healthy young individuals between 17 and 30 years of age (*M* = 22.1 *SD* = 3.1; 24 females, 25 highly educated) and 39 healthy older individuals between 64 and 90 years (age: *M* = 74.7 *SD* = 5.6; 23 females, 19 highly educated).

#### Procedure

In this study, participants performed 6 blocks of the Aperture Task. The experiment started with introductory (6 trials) and familiarizing trials (20 trials) for the active, button-pressing hand and subsequently, on the other hand. Afterward, participants performed each experimental block (32 trials) per hand.

#### Data analyses

Tests of normality with Shapiro-Wilk indicated that accuracy and detection values were not normally distributed within both groups (young: accuracy (active hand), Hit and False-Alarm rate (active and passive hand), criterion (c, active and passive hand): W(39) ≥ .789, *p* ≤ .041; older adults: False-Alarm rate, criterion (c, active and passive hand) W(39) ≥ .438, *p* ≤ .046). Therefore, group comparisons between young and older adults were run using non-parametric tests (Kruskal-Wallis-Test, Wilcoxon Test, Mann-Whitney U-Test). Corresponding z values reported by these tests were used to calculate the effect size *r* as proposed by Cohen [53] by dividing z by the square root of N (the procedure is also suggested by [54]). For pair-wise group comparisons (Mann-Whitney U-Test) statistical power (1-*β*; as the complement of Type II error magnitude) was computed by use of G*Power [55]. Test power was calculated two-tailed and with an alpha level of .05.

First, it was ensured that there was no effect of using the left vs. right hand for button presses between participants (young: *U* ≥ 121.5, *p* ≥ .054, *r* = .305; older adults: *U* ≥ 130.0, *p* ≥ .097, *r* = .270).

Data presented in the following results section are based on judgment performance in the experimental blocks separated according to active and passive hand.

### Study 1 results

Affordance judgment performance was compared for younger and older adults considering accuracy and signal detection values. We further analyzed DOSPERT questionnaire results on risk-perception and risk-taking for the health and safety subdomain as well as size perception.

#### Accuracy

On a descriptive level, older adults appeared to be less accurate. However, statistical comparisons (Table 1) revealed that younger and older adults did not differ significantly in their judgment accuracy.

**Table 1:** Descriptive data for young and older adults and between-subject group comparison results (Mann-Whitney U-Test).

		Young	Older		Group comparison		
Variable	Hand	*Mdn*	*Mdn*	*U*	*p*	1-*β*	*r*
Accuracy (%)	A	77.78	74.07	610.0	.132	.337	0.17
	P	74.07	74.07	571.0		.056	.580	0.22
Perceptual sensitivity (d’)	A	1.88	1.67	617.0		.153	.201	0.16
	P	1.68	1.67	634.5		.210	.360	0.14
Criterion (c)	A	-0.27	0.75	413.5		< .001	.980	0.39
P	0.06	0.84	398.5		< .001	.990	0.41
False-Alarm rate	A	0.17	0.04	402.0	< .001	.978	0.41
	P	0.17	0.04	402.0	< .001	.978	0.4
Hit rate	A	0.93	0.60	439.5	.001	.998	0.36
	P	0.80	0.53	415.0	< .001	.947	0.39

Power (1-*β*) was estimated two-tailed by using G*Power [55]. Effects of r can be interpreted based on [53]with the following intervals: r: .1 to .3: small effect; .3 to .5: intermediate effect; .5 and higher: strong effect. Mdn = median, A = active hand to be judged, P = passive hand to be judged.

#### Signal detection values

**Fig 2:**
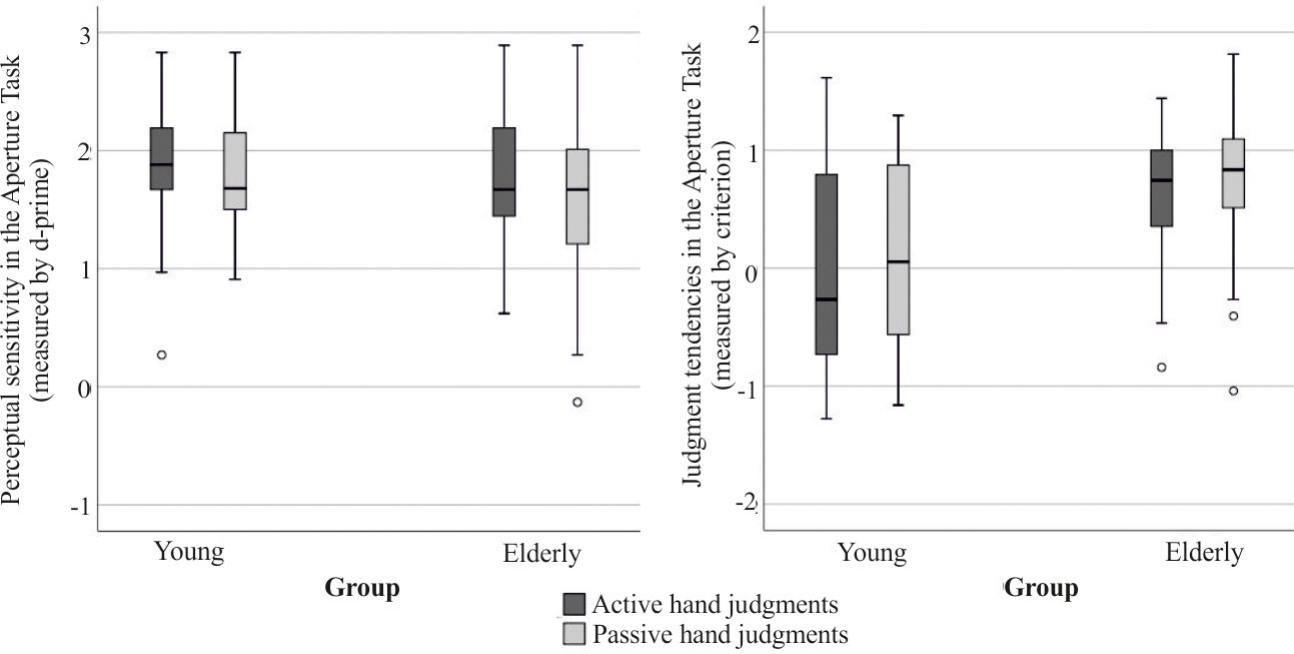
Boxplots for signal detection measures. Measures are displayed per group and hand to be judged. Older adults applied a more conservative judgment tendency compared to younger adults while demonstrating a similar level of perceptual sensitivity.

**Fig 3:**
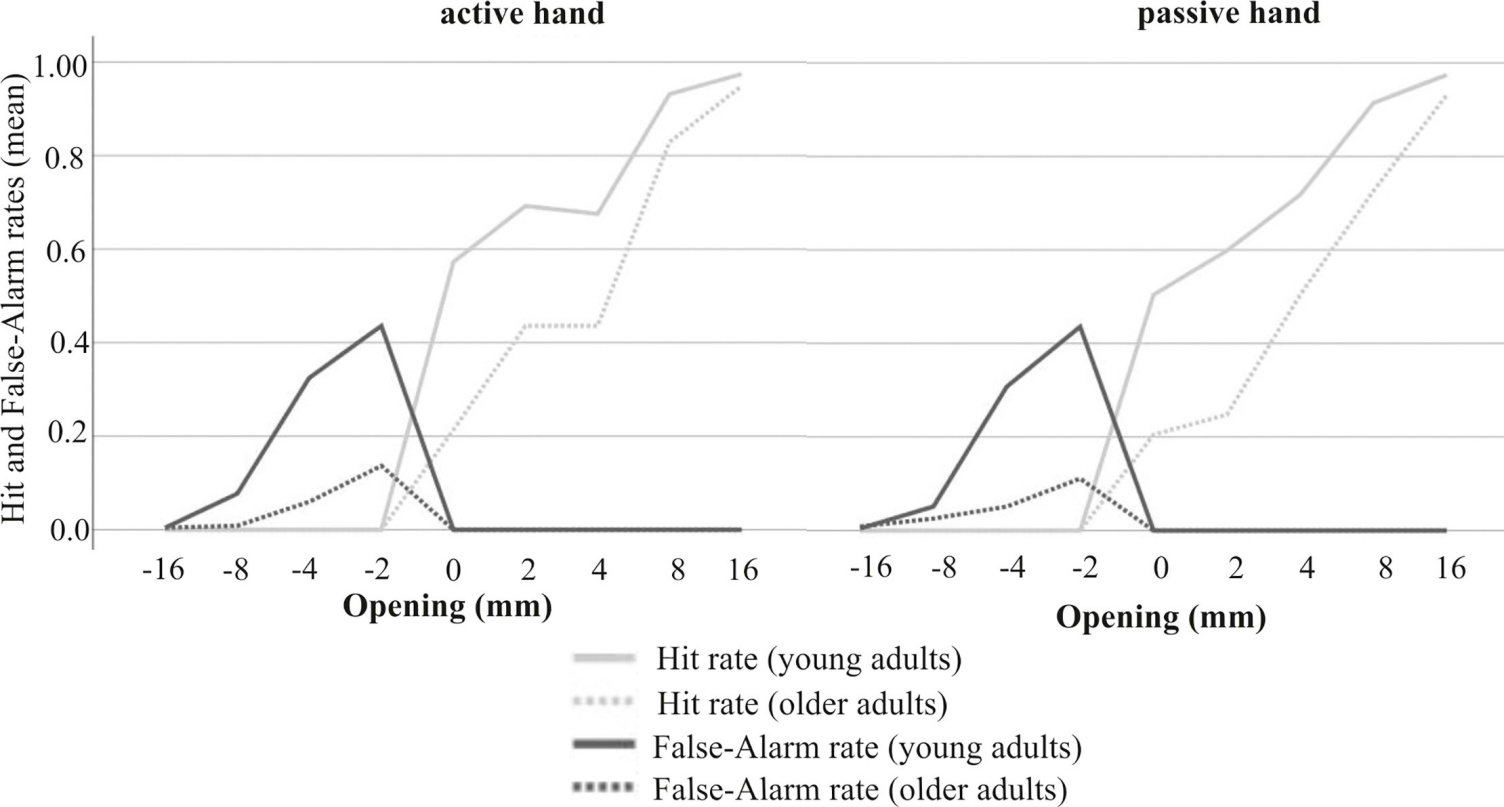
Hit and False-Alarm rates for younger and older adults. The figure displays an overview of changes in Hit- and False-Alarm rates for the different openings for the active and the passive hand, respectively. The opening “0” reflects a trial for which the respective hands just managed to fit in. Openings with negative mm-values were smaller (correct response: no), and those with positive values were larger than the hand’s individual fit (correct response: yes). The more conservative judgment tendency in older adults is indicated by a significantly lower Hit rate but also a significantly lower False-Alarm rate compared to younger adults.

Statistical comparisons (Table 1) revealed that the two groups did not differ regarding their ability to discriminate between a fit and a non-fit in the Aperture Task, but younger and older adults differed significantly regarding their produced judgment tendency. Accordingly, Boxplots in Fig 2 demonstrate that in contrast to younger adults, the older adults produced criterion values above 0, indicating rather conservative judgments. In order to shed more light on how subjects responded, we separately evaluated Hit and False-Alarm rates. Fig 3 displays the distribution of Hit and False-Alarm rates across the presented openings per group. The distribution across openings depicts that critical openings around zero are the most difficult to solve for either group. There was an increase in correct responses the greater the opening size deviated from zero with lower False-Alarm rates as well as higher Hit rates. Fig 3 further clearly demonstrates the differential behavior between groups and underlines the rather conservative judgment tendency chosen by older adults. Older adults produced a significantly lower Hit rate but also a significantly lower False-Alarm rate than younger adults (see also Table 1). This means that compared to younger adults, the older adults tended to respond with “no” more often in both types of events, in actual positive (correct responses) as well as actual negative ones (errors).

#### Risk-perception and risk-taking

Correlational analysis demonstrated that rather conservative judgment tendencies went along with higher risk-perception (*r*(61) ≥ .192, *p* ≤ .032) and lower risk-taking (*r*(61) ≤ -.158, p ≤ .077), whereby the latter did not reach significance. Further analyses concerning risk-taking and risk-perception behavior (health and safety DOSPERT subscales) demonstrated a significant effect of age. In line with their rather conservative judgment tendencies in the Aperture Task, older adults were less likely to engage in risky activities and concurrently rated the magnitude of these risks higher compared to younger participants (risk-taking: *U* = 111.5; *p* ≤ .001; risk-perception: *U* = 214.0; *p* ≤ .001).

#### Size estimation

Results from the size-estimation task revealed, that on average, younger adults overestimated hand size by 1.4 cm (*SD* = 0.95) and older adults by 2.2 cm (*SD* = 1.05). The mean difference between estimated and actual hand size correlates with the participants’ applied judgment tendency. Overestimating hand size went along with more conservative response tendencies in the Aperture Task (young: *r*(37) ≥ .495, *p* < .001; older adults: *r*(37) ≥ .247, *p* ≤ .03). Correlations between the perceptual sensitivity and the ability to accurately estimate hand size were not conclusive, since some (young: perceptual sensitivity_passive hand_: *r*(37) = -.282, *p* = .013; older: perceptual sensitivity_active hand_: *r*(37) = -.315, *p* = .006) but not all correlation coefficients revealed significance (young: perceptual sensitivity_active hand_: *r*(37) = —.138, *p* = .225; older: perceptual sensitivity_passive hand_: *r*(37) = —.206, *p* = .071).

### Study 1 discussion

In Study 1 we aimed at examining possible effects of advanced aging on actor-related affordance- judgments while also shedding light on specific aspects via use of the detection theory approach. Study 1 demonstrated that while healthy young and older adults used a significantly different response tendency, both groups’ performance appeared on a similar level in the affordance judgment task. These group differences in affordance judgment tendencies were mirrored by risk behavior evaluations delivered in health and safety statements in the DOSPERT questionnaire, both indicating that older adult participants appeared to be more cautious compared to the young adults.

In the applied Aperture Task, we found no general age decrements in older adults for discriminating a fit from a non-fit judgment and accuracy values did not differ significantly. These results are in line with other studies that also found a similar performance level for younger and older adults when judging whether they could squeeze their body through doorways [32] or selecting the greatest riser to stair climb [30]. Study 1 demonstrated that older adults chose a rather conservative response behavior, which may reflect a generally heightened concern about potential consequences of misjudgments and the perceived risk for harm. The health and safety statements delivered in the DOSPERT scales supported this interpretation. Here, older adults stated that they were less likely to engage in domain-specific risky activities and evaluated the magnitude of these risks generally to be higher. The current DOSPERT results on health and safety statements replicated the age-specific findings that were recently published by Bonem, Ellsworth [56]. To summarize, these findings suggest that older adults’ judgment behavior in the Aperture Task is guided by a more cautious judgment tendency which in contrast to younger adults is particularly reflected by avoiding False-Alarms and producing a lower Hit rate.

## Study 2

In Study 2, we aimed at examining whether participants would benefit from training and whether training effects would last over a one-week period. Each training session lasted approximately one and a half hours.

### Study 2 methods

#### Sample

Approximately half of the individuals from Study 1 also participated in Study 2; 20 healthy young individuals between 17 and 30 years of age (*M* = 22.5 *SD* = 3.9; 11 females, 12 highly educated) and 22 older individuals between 64 and 90 years of age (*M* = 74.27 *SD* = 6.4; 15 females, 10 highly educated).

#### Procedure

**Fig 4:**
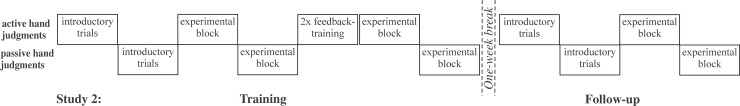
Experimental procedure in Study 2. In the experimental judgment blocks participants had to judge whether their hand may fit into a given opening. Only in feedback blocks participants had to actually try to fit their hand into the opening (training). One week after training a follow-up testing was conducted.

**Training Session.** Fig 4 provides an illustration of the procedure. On the second day of participation, the training session started with six introductory trials per hand, followed by one experimental block (32 trials) per hand assessing affordance judgments in the Aperture Task. Afterward, participants performed two training blocks (per block: 4x9 openings, plus 4 filler trials). Per training trial, participants first judged whether their hand could fit into the opening, and independent from their initial judgment, they subsequently were required to try to determine whether their flat hand (with fingers closely spaced) would fit. While fitting the hand through the opening, vision was provided. In case of a successful fit through, participants touched a backboard that was adjusted to hand-length distance and which included a sensor connected to a bell. By touching the board with their fingertip, the bell ring was triggered. Consequently, participants received acoustic, haptic and visual feedback. Acoustic feedback was only presented in trials for which the hand actually fitted through the opening. This acoustic feedback was delivered automatically by a bell. The bell was triggered when participants fitted into the aperture and touched a back-board. The back-board’s distance was adjusted to the individual’s hand length. This supported correct interpretation of fits vs. non-fits since feedback was only delivered in case of a complete and successful fit through.

In future anticipation of applying this training paradigm to stroke patients with hemiplegia who would not be able to use their paretic arm, training blocks were only conducted for the assigned active button pressing hand. Thus, the passive hand remained untrained. After the two training blocks, participants performed one experimental block with each hand, starting with the active hand. Overall, participants completed 8 blocks within this session: 5 blocks with judgments for the active hand and 3 blocks with judgments for the passive hand.

**Follow-up Session.** In order to examine whether training effects are lasting, a follow-up session with 4 block trials took place one week later (between 5 and 7 days after training). The experiment started with introductory trials for the active hand and subsequently for the passive hand. Afterward, participants performed one experimental block per hand.

#### Data analyses

In addition to signal detection values, we included again judgment accuracy (percent of correct judgments) in order to evaluate the training effect in detail. Tests of normality indicated that signal detection values (Hit rate, False-Alarm rate, perceptual sensitivity, criterion c) and judgment accuracy values were not normally distributed in at least one group (separated for training and follow-up session as well as for judgments upon the active or passive hand). Again, there was no significant difference between participants who used the left or right hand for button presses within the respective age group (young: *U* ≥ 34.5, *p* ≥ .255, older adults: *U* ≥ 41.0, *p* ≥ .220; please note that there was one exception in older adults: False-Alarm rate (passive hand, pre-training): *U* = 33.5.0, *p* = .035. *r* = .373). Based on our stated hypotheses, we ran a Friedman Test to evaluate whether there was a main effect of session (pre-training, post-training, follow-up) within groups and per variable (accuracy and signal detection data, separated for hand to be judged). Based on significant results, the Wilcoxon Test was run to evaluate the predicted effect of training (pre-training judgments versus post-training judgments) as well as training sustainability (pre-training judgments versus follow-up judgments). The calculation of the effect size r was based on z values reported by the Wilcoxon Test [53]. Statistical power (1-β) for these comparisons of matched pairs was computed by use of G*Power [55]. Test power was calculated two-tailed and with an alpha level of .05. Judgments for the active versus the passive hand were considered separately from one another since only the active hand had been trained. Furthermore, this provides information on whether there is a generalized training effect on judgments for the non-trained (passive) hand.

### Study 2 results

First, we analyzed whether the sole repetition of the Aperture Task influenced judgment performance. Neither young nor older adults improved solely due to repeated task execution when comparing initial performance (perceptual sensitivity, judgment tendency) in the experimental session of Study 1 with pre-training performance in the first session of Study 2 (young: *Z* ≤ -0.40, *p* ≥ .329, *r* ≥ .089; older adults: *Z* ≤ 0.0, *p* ≥ .218, *r* ≥ .0).

To assess trainability, in Study 2 participants received training for the assigned active button pressing hand. Performance was evaluated pre-training, during training, post-training and for the follow-up after one week. For a descriptive overview and test statistics see Table 2. In the following, we will describe significant results per variable.

**Table 2:** Descriptive data for young and older adults as well as post-hoc analyses comparing pre-training performance with post-training as well as follow-up performance (Wilcoxon-Tests, only listed for variables with a significant effect of session indicated by Friedman).

			Pre-Training		Post-Training					Follow-up			
	Variable	Hand	*Mdn* *[IQR]*	*Mdn* *[IQR]*	*Z*	*p*	1-*β*	*r*	*Mdn*	*Z*	*p*	1-*β*	*r*
Young	Acc (%)	A	77.78 [70.37, 81.48]	87.04 [78.70, 91.67]	-3.48	< .001	.997	.777	81.48 [77.78, 85.19]	-2.17	.030	.636	.486
		P	79.63 [70.37, 85.19]		81.48 [77.78, 85.19]	-	-		-	77.78 [74.07, 88.89]	-	-		-
	d-prime	A	1.84 [1.50, 1.98]	2.38 [1.91, 2.76]	-3.21	.001	.979	.718		2.04 [1.82, 2.25]	-2.09	.036	.640	.468
		P	1.88 [1.67, 2.35]	2.05 [1.82, 2.29]	-	-		-	1.88 [1.63, 2.54]	-	-		
	criterion	A	0.44 [-0.76, 0.92]	-0.14 [-0.63, 0.14]	-	-		-	0.01 [-0.45, 0.64]	-	-		-
		P	0.49 [-0.60, 0.90]	-0.25 [-0.54, 0.58]	-	-		-	0.14 [-0.42, 0.75]	-	-		-
	FA rate	A	0.08 [0.04, 0.40]	0.13 [0.08, 0.33]	-	-		-	0.13 [0.05, 0.25]	-	-		-
		P	0.08 [0.04, 0.31]	0.17 [0.05, 0.31]	-	-		-	0.08 [0.04, 0.29]	-	-		-
	Hit rate	A	0.67 [0.47, 0.96]	0.93 [0.87, 0.97]	-3.22	< .001	.956	.719	0.87 [0.62, 0.96]	-1.57	.120	.333	.506
		P	0.73 [0.47, 0.97]	0.87 [0.62, 0.96]	-	-		-	0.87 [0.57, 0.93]	-	-		-
Older	Acc (%)	A	77.78 [64.82, 86.11]	85.18 [77.78, 89.82]	-2.40	.015	.668	.511	81.48 [70.37, 88.89]	-1.44	.158	.726	.307
		P	74.07 [62.04, 82.41]	81.48 [76.85, 85.19]	-2.66	.006	.811	.567	83.33 [77.78, 88.89]	-2.51	.011	.249	.534
	d-prime	A	1.93 [1.41, 2.39]	2.16 [1.77, 2.61]	-	-		-	2.04 [1.63, 2.54]	-	-		-
		P	1.62 [1.27, 2.21]	1.92 [1.76, 2.36]	-	-		-	2.15 [1.81, 2.43]	-	-		-
	c	A	0.75 [0.31, 1.05]	0.14 [-0.47, 0.51]	-3.23	.001	.912	.689	0.27 [-0.31, 0.86]	-3.10	.001	.748	.661
		P	0.83 [0.37, 1.12]	-0.02 [-0.48, 0.59]	-3.77	< .001	.972	.804	0.47 [-0.27, 0.75]	-2.99	.002	.768	.637
	FA rate	A	0.04 [0.04, 0.05]	0.08 [0.04, 0.25]	-2.58	.007	.714	.550	0.08 [0.04, 0.25]	-2.51	.012	.956	.534
		P	0.04 [0.04, 0.08]	0.17 [0.04, 0.27]	-3.18	< .001	.802	.678	0.06 [0.04, 0.17]	-2.10	.039	.322	.448
	Hit rate	A	0.60 [0.37, 0.87]	0.87 [0.73, 0.93]	-3.05	.001	.929	.650	0.80 [0.47, 0.97]	-2.38	.015	.603	.506
		P	0.53 [0.32, 0.75]	0.87 [0.72, 0.93]	-3.78	< .001	.982	.807	0.73 [0.60, 0.93]	-3.07	.001	.848	.654

Power (1-β) was estimated two-tailed by using G*Power [55]. Effects of r can be interpreted based on [53] with the following intervals: r: .1 to .3: small effect; .3 to .5: intermediate effect; .5 and higher: strong effect. Mdn = median, IQR = interquartile range, A = active hand to be judged, P = passive hand to be judged.

#### Accuracy

The results of judgment accuracy (percent of correct judgments) demonstrated that most participants improved their judgments immediately after training.

Friedman Test results revealed a main effect of session (pre-training, post-training, follow-up) for judgment accuracy in younger (active hand: χ^2^(2) = 13.162, *p* = .001) and *older* adults (active and passive hand: χ^2^(2) ≥ 6.975, *p* ≤ .028). Table 2 lists post hoc analyses comparing the performance in the pre-training session with performance immediately after practice as well as with performance after a one-week break. In younger participants, pre-training judgment accuracy values for the active hand significantly differed from values immediately after practice as well as after a one-week break. In older adults, pre-training judgment accuracy values for the active as well as the passive hand significantly differed from the post-training session. For the passive hand a significant training effect was still apparent one week after practice.

Significant training effects were found with effect sizes ≥ .5 ([53]; see Table 2). More than 40 percent of the participants demonstrated more than 10 percent improvement (active hand judgments: 45% older adults, 65% young adults; passive hand judgments: 41% older adults, 45% younger adults). After a one-week break, more than 30% of the participants still demonstrated improved performance by achieving a 10 percent accuracy increase compared to pre-training (active hand judgments: 32% older adults, 35% young adults; passive hand judgments: 41% older adults, 30% young adults). Despite the significant training effects on a group level, inter-individual differences in trainability need to be acknowledged. A few participants in both age groups did not benefit from training when judging their trained hand immediately after training (young: *n* = 2, older: *n* = 5).

#### Perceptual sensitivity

**Fig 5:**
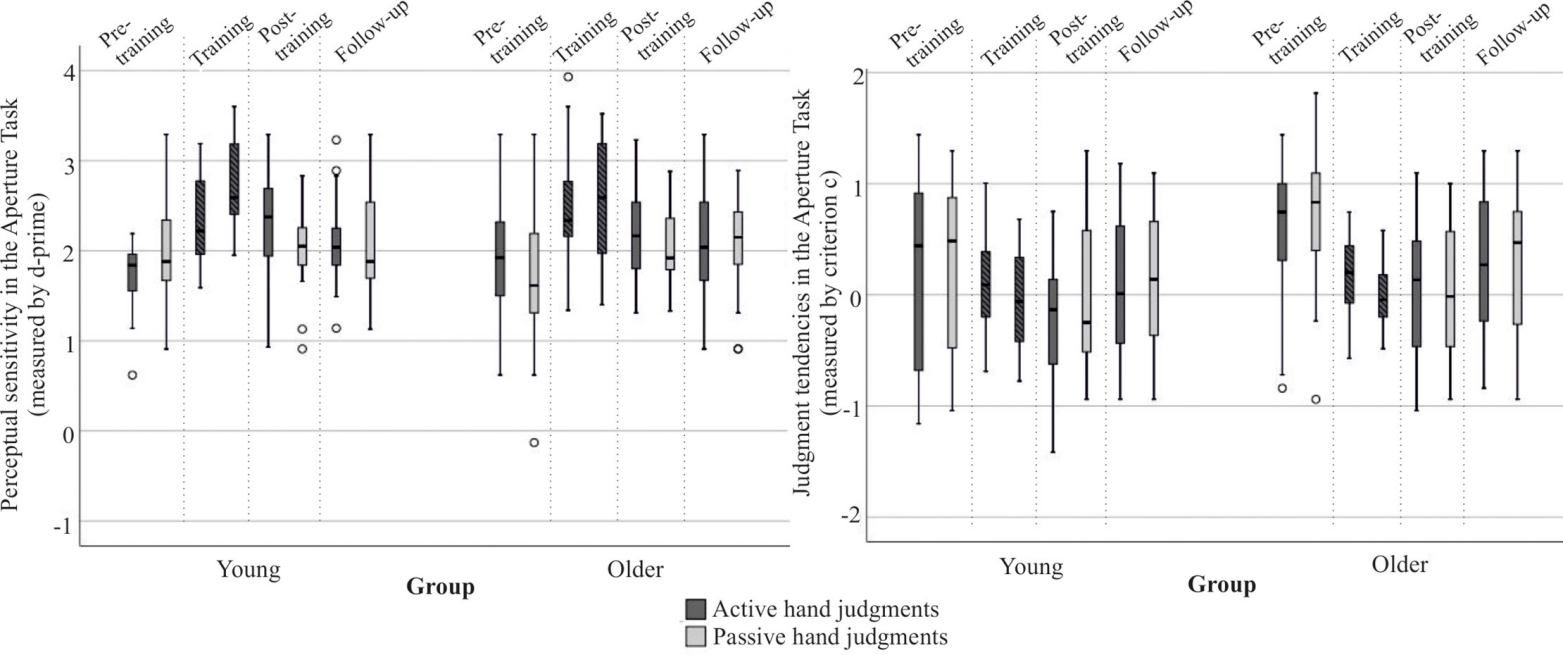
Performance in perceptual sensitivity and judgment tendencies in young and older adults before, during and after training. (A) Boxplots indicate perceptual sensitivity for discriminating a fit from a non-fit each for the active and passive hand. (B) Boxplots display judgment tendencies per hand to be judged. Please note, participants received training for the assigned button-pressing active hand only.

In younger adults, Friedman Test results revealed a significant main effect of session (pre-training versus post-training versus follow-up) for perceptual sensitivity judging the active hand (χ^2^*(*2) = 10.95, *p* = .003). Further post hoc comparisons demonstrated that younger adults significantly improved judgments for their active hand immediately after practice and after a one-week break as compared to pre-training performance. Older adults failed to achieve a significant session effect in this performance measure for either hand (*p* ≥ .091). The descriptive statistics demonstrated that perceptual sensitivity was quite varied in older adults in the pre-training measurement (perceptual sensitivity for active hand: .62–3.29 and passive hand: -.13–3.29), but immediately after training a trend towards reduced variability and improved values in perceptual sensitivity became apparent (for the active hand: 1.31–3.23 and passive hand: 1.33–2.88) (see medians in Table 2 and boxplots in Fig 4). Boxplots in Fig 5 indicate that both groups demonstrated the best performances in discriminating a fit from a non-fit within training blocks (Fig 5A). A change in behavior during the training intervention was also reflected in adjustments of judgment tendencies, which on a group-level appeared almost perfectly close to 0 in both groups. In post-training and follow-up sessions, the median criterion values revealed less conservative judgment tendencies compared to pre-training for both groups.

#### Hit and False-Alarm rates

**Fig 6:**
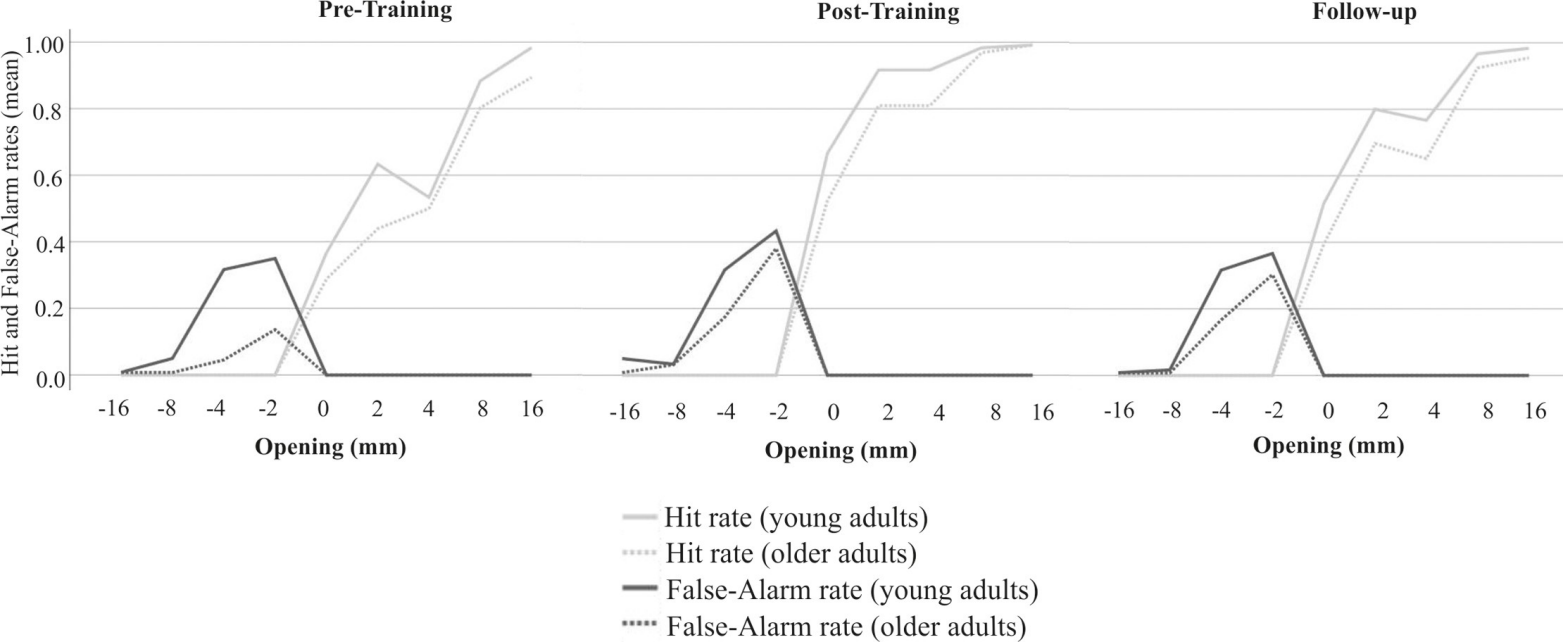
Hit and False-Alarm rates for younger and older adults before and after training. The figure displays an overview of changes in Hit- and False-Alarm rates for the trained hand (button-pressing active hand). The x-axis lists the different aperture openings related to the actual hand width (0). Compared to the individual’s hand width, positive and negative increments reflect larger (correct response: yes) or smaller (correct response: no) openings respectively. Training led to an increase in yes-responses going along with increases in Hits and False Alarm rates.

To provide a more thorough understanding of how training had affected participants’ judgment behavior, we further considered Hit and False-Alarm rates. In younger adults, Friedman Test results revealed a significant main effect of session (pre-training versus post-training versus follow-up) only for Hit rates with the active hand (χ^2^*(*2) = 7.19, *p* = .027). Post hoc comparisons revealed that there was a significant increase in the Hit rate immediately after practice. In older participants, Friedmann Test results indicated a significant session effect (pre-training versus post-training versus follow-up) in Hit and False-Alarm rates with respect to both hands (χ^2^*(*2) ≥ 10.47, *p* ≤ .004). Hit and False-Alarm rate changed significantly in the long-term due to training (Table 2). Active hand responses are depicted in Fig 6 illustrating the shifts in Hit- and False-Alarm rates across the different aperture openings before and after training.

#### Judgment tendencies

Friedman Test results revealed an effect of session (pre-training versus post-training versus follow-up) on judgment tendencies in older adults (χ^2^ (2) ≥ 13.310, *p* ≤ .001). Post hoc comparisons revealed that in post-training as well as in the follow-up, older adult judgment tendencies for the active, as well as for the passive hand, were significantly less conservative compared to the pre-training session. Since the Friedmann Test results revealed no significant training effects for younger adults (*p* ≥ .187), we refrained from further pairwise comparisons.

### Study 2 discussion

Previous work with younger adults indicated that affordance judgments can be improved by training [15, 20, 57]. In the current study, we have shown that training within one session was sufficient to adjust actor-related affordance judgments in an Aperture Task in young and older adults. Improvements were present during and after training, irrespective of whether the hand to be judged was the actually trained hand. Improvements were still present after a one-week follow-up. Detection theory analysis demonstrated that the two age groups improved in different aspects of affordance judgments. While young adults were able to enhance their level of perceptual sensitivity by increasing their Hit rate, older adults adjusted their judgment tendency towards less conservative judgments by increasing both Hit- and the False-Alarm rates. We here preferred to use the negative description of ‘less conservative’ instead of the term ‘liberal’, because older adults on average have a criterion value larger than zero which indicates a conservative judgment behavior. These results emphasize the potential benefits of elucidating different aspects of performing affordance judgments. Differentiating the quality of improved performance may help to evaluate the benefits or weaknesses of certain types of settings and training. For example, the question arises whether a significant increase in False-Alarm rates is indeed acceptable in certain settings. Future studies should attempt to define training settings and conditions that provide the best benefits for affordance judgments.

## General discussion

Actor-related affordance judgments are decisions concerning the outcome of an effective and efficient fit between our bodily actions and the environment, for example, when judging whether that tea-cup on the shelf is within reach, or whether one may be able to squeeze the hand into the locked mailbox to retrieve a letter. These judgments are thought to be substantially influenced by the comparison of on-line perceived environmental properties with an experience-based judgment criterion. Because the actor’s bodily and cognitive capabilities typically alter due to aging, these changes need to be taken into account by the actor when judging affordances.

With the current work, we aimed at further elucidating the effects of advanced aging on affordance judgments and particularly the trainability of this ability by use of an Aperture Task. In two studies, we examined young and older adults who judged whether their hands may fit into a presented opening. Next, to analyze the percentage of accurate responses as an indicator of judgment performance, we used a detection theory approach to evaluate characteristics such as perceptual sensitivity and judgment tendency. Overall, Study 1 demonstrated that older adults compared to younger adults were more concerned about the potential consequences of misjudgments and applied a more conservative judgment tendency, while the perceptual sensitivity did not differ significantly between groups. More conservative judgments in the Aperture Task seemed to go along with stronger overestimations of the perceived individual’s hand size. In Study 2, training in the Aperture Task led to significant increases in accuracy even after a one-week follow-up. While younger adults improved in perceptual sensitivity, older adults adjusted their tendency towards less conservative judgments. However, any potential task-independent changes in judgment tendency or even risk perception due to the intervention had not been measured, i.e. the DOSPERT scale was only administered once at the beginning of the study.

Too conservative or liberal decisions might have limiting effects on independence as well as self-awareness in older adulthood. That is why there is a general need for accurate affordance judgments that are neither distorted in a conservative nor a liberal direction. Exemplified by the more frequent use of wheelchairs or walkers in older adulthood, the need for accurate affordance judgments becomes apparent. In order to benefit from mobility aids and to regain autonomous mobility, older adults have to adapt their affordance judgments and update their references regarding their altered action capabilities and constraints.

Therefore, perhaps the most promising finding of this work is the trainability of the implemented affordance judgment task that appeared effective even in older adults with one session leading to measurable behavioral changes one week later. However, future studies need to closely monitor the quality of such behavioral changes and the resulting benefits as well as potential disadvantages. Indeed, we observed a significant improvement in accuracy and an adjustment of conservative judgment tendency towards a rather balanced judgment tendency in older adults. However, while their behavioral adjustment went along with a significant increase in Hits, it also led to higher False-Alarm rates.

Clearly, the present study is still only a start unraveling the underlying mechanisms of the trainability of affordance judgments. Nevertheless, our findings are in line with more recent affordance models from cognitive neuroscience that suggest a large bilateral brain network being involved in affordance judgments, representing a complex interplay of different and dynamic motor cognitive skills [37–39]. The underlying assumption of these dynamic models is the general ecological benefit of a flexible system including parallel recruitment of various brain processes while making affordance judgments (from visuospatial attention to biasing action selection). The ecological concept has its origins in Gibson’s classical affordance theory. This ecological approach predicts that performance in affordance judgments is task specific (this has further been substantiated by experimental studies as e.g. [17, 21]). We propose that dependent on the respective task and setting, crucial functional components in the system may receive more processing weights compared to other components loading less on a specific task. For instance, rigid fitting tasks (e.g., Aperture Task) are predominantly based on information about rather stable bodily properties (e.g., hand or shoulder width) and aperture size. Depending on task instructions, fitting tasks can be also less rigid for example by introducing additional degrees of freedoms when e.g. squeezing of the hand [14] or shoulder rotation [58] is possible and allowed. Reaching tasks might introduce even more degrees of freedom including more variable bodily properties (e.g., arm length, distance and angles). Accordingly, based on the assumption that affordance judgment performance is task specific, it may only be trainable on a task-specific level. Consequently, additional studies are required to evaluate the trainability of judgments in other affordance judgment tasks. Thereby sample specific characteristics need to be considered. There are several tasks that afford actions that might be limited in their execution with advancing age and demand for adaption of judgments due to age-related bodily changes (e.g., descending stairs, stepping across obstacles, reaching for objects).

## Conclusions

The current study investigated effects of aging in affordance judgments by use of an Aperture Task. When judging whether or not a hand fits into a slot, older compared to younger adults did not differ in perceptual sensitivity. Yet, older adults produced rather conservative judgments, and they appeared to have a heightened concern about potential consequences of misjudgments.

It seems promising that affordance judgments in young and older adults were trainable within one training session, and that these training effects were present even one week later. However, whether in the long run our aging society may benefit from training batteries improving the skill of affordance judgments needs to be shown. Fundamental studies on affordance judgments are still needed to further elucidate the complex interplay of different aspects of this motor cognitive skill. Conditions of trainability need to be enlightened across different task demands while considering the remarkable attributes and attitudes that are specific for a tested sample, such as older age and heightened risk perception.
