# Correction: Does it fit?—Trainability of affordance judgments in young and older adults

**DOI:** 10.1371/journal.pone.0215438

**Published:** 2019-04-10

**Authors:** Lisa Finkel, Simone Engler, Jennifer Randerath

The calculation of the effect sizes in Study 2 is incorrect. The denominator N should reflect the number of observations (number of participants * number of measures) and not sample size, as previously used in the analysis.

In the Study 2 results subsection of Study 2, there is an error in the last sentence of the first paragraph. The correct sentence is: Neither young nor older adults improved solely due to repeated task execution when comparing initial performance (perceptual sensitivity, judgment tendency) in the experimental session of Study 1 with pre-training performance in the first session of Study 2 (young: Z ≤ -0.40, p ≥ .329, r ≥ .063; older adults: Z ≤ 0.0, p ≥ .218, r ≥ .0).

In the Study 2 results subsection of Study 2, there is an error in the first sentence of the fifth paragraph. The correct sentence is: Significant training effects were found with effect sizes ≥ .3 ([53]; see Table 2).

In Table 2, the column with reported effect size values are incorrect. Please see the correct Table 2 here.

**Table 2:** Descriptive data for young and older adults as well as post-hoc analyses comparing pre-training performance with post-training as well as follow-up performance (Wilcoxon-Tests, only listed for variables with a significant effect of session indicated by Friedman.

			Pre-Training	Post-Training					Follow-up				
	Variable	Hand	*Mdn* *[IQR]*	*Mdn* *[IQR]*	*Z*	*p*	1-*β*	*r*	*Mdn*	*Z*	*p*	1-*β*	*r*
Young	Acc (%)	A	77.78 [70.37, 81.48]	87.04 [78.70, 91.67]	-3.48	< .001	.997	.550	81.48 [77.78, 85.19]	-2.17	.030	.636	.343
		P	79.63 [70.37, 85.19]	81.48 [77.78, 85.19]	-	-	-	-	77.78 [74.07, 88.89]	-	-	-	-
	d-prime	A	1.84 [1.50, 1.98]	2.38 [1.91, 2.76]	-3.21	.001	.979	.508	2.04 [1.82, 2.25]	-2.09	.036	.640	.330
		P	1.88 [1.67, 2.35]	2.05 [1.82, 2.29]	-	-	-	-	1.88 [1.63, 2.54]	-	-	-	-
	criterion	A	0.44 [-0.76, 0.92]	-0.14 [-0.63, 0.14]	-	-	-	-	0.01 [-0.45, 0.64]	-	-	-	-
		P	0.49 [-0.60, 0.90]	-0.25 [-0.54, 0.58]	-	-	-	-	0.14 [-0.42, 0.75]	-	-	-	-
	FA rate	A	0.08 [0.04, 0.40]	0.13 [0.08, 0.33]	-	-	-	-	0.13 [0.05, 0.25]	-	-	-	-
		P	0.08 [0.04, 0.31]	0.17 [0.05, 0.31]	-	-	-	-	0.08 [0.04, 0.29]	-	-	-	-
	Hit rate	A	0.67 [0.47, 0.96]	0.93 [0.87, 0.97]	-3.22	< .001	.956	.509	0.87 [0.62, 0.96]	-1.57	.120	.333	.248
		P	0.73 [0.47, 0.97]	0.87 [0.62, 0.96]	-	-	-	-	0.87 [0.57, 0.93]	-	-	-	-
Older	Acc (%)	A	77.78 [64.82, 86.11]	85.18 [77.78, 89.82]	-2.40	.015	.668	.362	81.48 [70.37, 88.89]	-1.44	.158	.726	.217
		P	74.07 [62.04, 82.41]	81.48 [76.85, 85.19]	-2.66	.006	.811	.401	83.33 [77.78, 88.89]	-2.51	.011	.249	.378
	d-prime	A	1.93 [1.41, 2.39]	2.16 [1.77, 2.61]	-	-	-	-	2.04 [1.63, 2.54]	-	-	-	-
		P	1.62 [1.27, 2.21]	1.92 [1.76, 2.36]	-	-	-	-	2.15 [1.81, 2.43]	-	-	-	-
	c	A	0.75 [0.31, 1.05]	0.14 [-0.47, 0.51]	-3.23	.001	.912	.487	0.27 [-0.31, 0.86]	-3.10	.001	.748	.467
		P	0.83 [0.37, 1.12]	-0.02 [-0.48, 0.59]	-3.77	< .001	.972	.568	0.47 [-0.27, 0.75]	-2.99	.002	.768	.451
	FA rate	A	0.04 [0.04, 0.05]	0.08 [0.04, 0.25]	-2.58	.007	.714	.389	0.08 [0.04, 0.25]	-2.51	.012	.956	.378
		P	0.04 [0.04, 0.08]	0.17 [0.04, 0.27]	-3.18	< .001	.802	.479	0.06 [0.04, 0.17]	-2.10	.039	.322	.317
	Hit rate	A	0.60 [0.37, 0.87]	0.87 [0.73, 0.93]	-3.05	.001	.929	.460	0.80 [0.47, 0.97]	-2.38	.015	.603	.359
		P	0.53 [0.32, 0.75]	0.87 [0.72, 0.93]	-3.78	< .001	.982	.570	0.73 [0.60, 0.93]	-3.07	.001	.848	.463

Power (1-β) was estimated two-tailed by using G*Power [55]. Effects of r can be interpreted based on [53] with the following intervals: r: .1 to .3: small effect; .3 to .5: intermediate effect; .5 and higher: strong effect. Mdn = median, IQR = interquartile range, A = active hand to be judged, P = passive hand to be judged.

## References

[1] Finkel L, Engler S, Randerath J (2019) Does it fit?–Trainability of affordance judgments in young and older adults. PLoS ONE 14(2): e0212709 10.1371/journal.pone.0212709 30817755 PMC6395027

